# “*Infeliz”* or “*Triste”*: A Paradigm for Mixed Methods Exploration of Outcome Measures Adaptation Across Language Variants

**DOI:** 10.3389/fpsyg.2021.695893

**Published:** 2021-08-09

**Authors:** Chris Evans, Clara Paz, Guido Mascialino

**Affiliations:** ^1^Department of Psychology, The University of Sheffield, Sheffield, United Kingdom; ^2^Escuela de Psicología, Universidad de Las Américas, Quito, Ecuador

**Keywords:** cross-cultural adaptation, outcome measures, translations, mixed methods, research methodology, CORE-OM (Clinical Outcomes in Routine Evaluation—Outcome Measure), recommendations

## Abstract

The literature on measure translation tends to hold, overtly or covertly, a questionable assumption about the possibility of exact translation and almost completely ignores issues of within language variation. Equally, psychometric methods used to assess cross-cultural validity after translation focus on large sample tests of cross-sectional measurement invariance. Such invariance is often not found and is of dubious pertinence to change/outcome measures usually used in psychotherapy research. We present a sequential process of three substudies using quantitative and qualitative procedures to explore whether an outcome measure needs to be changed when used across language variation. Qualitative data confirmed that an item was not ideal in the new context. However, quantitative exploration showed that, although statistically significant and affected by gender and item order, the impact of changing the item in the overall score was small, allowing retention of the existing Spanish translation. We argue that the myth of perfect translation and over-reliance on large-sample psychometric testing pursuing measurement invariance limit exploration of language effects. We recommend that these be used in the companion of user-based, sequential, mixed-method exploration to support the development of a richer field of understanding of outcomes and change self-report measures across languages and cultures and both across and within languages.

## Introduction

Self-report questionnaires are frequently used in psychology to explore constructs that cannot be measured directly (Buntins et al., 2016); in psychotherapy, we use such measures of problems, symptoms, well-being and quality of life (Tarescavage and Ben-Porath, 2014). Scores from these measures are used to understand change across therapy interventions. The use of these measures has extended in the last decades, and some of them are now offered in contexts markedly different from those where they were created. Scores derived from these self-reported questionnaires are not like blood test results (Paz et al., 2020a) but shaped by individual (Truijens et al., 2019), cultural (Evans et al., 1997), and, certainly, linguistic issues (Harzing, 2005).

Most self-report questionnaires are created in developed countries and typically the original version is in English. Often, successful measures go on to be translated and used in other countries. Translating an existing measure is faster and cheaper than generating a new one, and the hope is that scores on the translated measure can be compared with those from the existing instrument (Hambleton and Patsula, 1999). There are more than 30 guidelines about cross-cultural adaptation (CCA) of questionnaires, yet there is no consensus about which one is the best methodology (Epstein et al., 2015b), probably because there is no one perfect method any more than there are perfect translations. Studies showing empirical comparison between methods illustrate the challenges one would face in trying to develop a “gold standard” (Perneger et al., 1999; Hagell et al., 2010; Epstein et al., 2015a) and should probably be understood to reject that idea. Most modern guidelines were developed on experience of researchers (Guillemin et al., 1993; Beaton et al., 2000; Hambleton and De Jong, 2003; International Test Commission, 2018) and included several steps, beginning generally with multiple initial translations, synthesis/reconciliation of translations, back-translation, expert committee review, and, sometimes, ending with forms of “pretesting” (Sartorius and Kuyken, 1994; Beaton et al., 2000; Epstein et al., 2015b), i.e., pilot studies exploring basic psychometric properties typically of internal reliability and/or factor structure, in order to spot problems and make revisions. Of note, most guidelines pay little attention to what should happen during the “pretesting” stage, particularly when a translation has satisfactorily been completed (Beaton et al., 2000; Hambleton and De Jong, 2003; Epstein et al., 2015b). As a result, measures may move into the full-scale psychometric exploration phase before they are adequately translated, and problems with the instrument only become evident after large-scale “validation” studies. Consequently, these guidelines risk divorcing translation from psychometric exploration.

Currently, there is a large body of literature covering cross-cultural psychometric exploration of measures, with several journals addressing it specifically (e.g., Journal of Cross-Cultural Psychology), or as a recurring theme within more general methodological focus (e.g., Psychology, Psychological Assessment, Psychological Methods). That work typically focuses on seeking “measurement invariance” across cultural groups (Vandenberg and Lance, 2000; Byrne and Watkins, 2003; Milfont and Fischer, 2010), using either classical test theory (CTT), typically confirmatory factor analysis (CFA), or item response theory (IRT) methods. These psychometric approaches are powerful, and, where the assumptions they make are sound, they become sensible ways to develop cross-cultural measures that compare performance of individuals (e.g., to determine entry to schools or universities). However, there is literature going back certainly to the late 20th century showing that almost none of these forms of measurement invariance are found for pragmatic mental health change measures, even within one language (Kim et al., 2010; Fried et al., 2016), which suggests that alternative forms of testing equivalence between versions should be considered (e.g., Paz et al., 2021).

Translation guidelines and cross-cultural psychometric research also have very little to say about use of measures in two different locations sharing the same language but having clear cultural differences. Guillemin et al. (1993) make a passing reference to this situation, and they recommend an expert-based approach, through a review committee, to adapt the measure to the culture of the target population. However, no specific actions are presented of how to conduct and test this process. This is not because the issue is unimportant or rare: a number of languages are spoken in several countries, even in different continents, each with disparate cultural contexts and distinctive economic and political situations that can impact the levels of literacy, concordance between spoken and written language, social desirability, and presence of taboo subjects and other issues (Sartorius and Kuyken, 1994). Languages such as English, Arabic, French, Portuguese, and Spanish, are spoken across the globe, yet their characteristics, such as lexicon, spelling, grammar and phraseology, vary widely. Spanish, for example, originated in a small central northern region of the Iberian peninsula and before becoming one of the world's most widely-spoken languages, underwent numerous transformations, including and crucially ones resulting from the colonization of the Americas (Erker, 2017). The specialization of Spanish into several Latin American regional variants was a prolonged and complex process for various reasons such as contact with indigenous languages, regional differences in Spanish dialects among the arriving Spaniards, and, in some cases, notably Argentina and Uruguay (Lipski, 2014), mass immigration in the twentieth century from various countries further affecting the use of Spanish language. These factors generated linguistic changes significant enough to be termed “language varieties” by linguists and are strong enough to be represented in language-coding schemes for information management, such as library organization and/or software development (Penix-Tadsen, 2016; Gutiérrez-Artacho and Olvera-Lobo, 2017). Despite these coding systems, and recognition of language variation by linguists, we could not find literature addressing the intricacies of within-language varieties in the adaptation of mental health questionnaire measures. Where language variation is recognized, checks on questionnaire fit to the local context are clearly necessary, but the challenge is different from that of translation of measures between languages. Without translation, a measure will be simply unusable for most members of the target population. By contrast, failure to consider language variation risks wide use of a measure but without recognition that, despite the same translation being used, answers from different locations may not be comparable because of language variation.

This language variant issue was the setting of the research question: what was the empirical evidence that a measure already translated from English to Spanish, translated in Spain, might need another translation or adaptation for use in another Spanish-speaking country, specifically Ecuador? The literatures noted above, on translation of measures and “cross-cultural validation,” seemed to offer us little guidance. This study reports three sequential empirical studies addressing the question and explains the decisions we made in light of the findings. The purpose of presenting in detail each study is to provide enough information on some methodological alternatives that dissect language and cultural effects using a series of qualitative and quantitative explorations during a pre-testing stage, rather than a single large cross-sectional “validation” survey once the measure is considered finalized. As we knew, there is some language variation across Spanish within Ecuador; this too was checked, although expected to have a small impact.

The measure used in this exploration is the Clinical Outcomes in Routine Evaluation-Outcome Measure (CORE-OM; Evans et al., 2002), which is widely used in psychotherapy research to track psychological distress. The CORE-OM was selected, because it is a “copyleft” measure, i.e., illegal to change but free to reproduce, crucial for use in countries where resources for research and generation of evidence related to mental health are scarce. The CORE-OM has been translated, to the CORE translation requirements, into more than 25 languages (CORE System Trust, 2015) such as Spanish (Trujillo et al., 2016); the most recent study gives a very detailed exploration of how cultural and linguistic issues are handled in CORE translations (Yassin and Evans, 2021).

In the original Spanish translation of the CORE-OM (Trujillo et al., 2016), 10 people (6 psychology professionals and 4 lay people) were asked to translate the English CORE-OM into Spanish. A group composed of two psychology professionals, two lay people, and a member of the CORE System Trust (https://www.coresystemtrust.org.uk/) formed an expert panel and reached a consensus regarding the best translation for each item. The final version was reviewed by three experts in psychology, who suggested modifications, which were discussed with the member of the CORE System Trust. Once completed, the version was pre-tested with a group of 64 people who also give feedback about understanding and appropriateness of each item. All these comments were discussed by a group of experts and a new version was construed. This version was back-translated, which suggested that no further modifications were needed. The psychometric properties of the scores in the Spanish sample (Trujillo et al., 2016) were broadly comparable with those found for the scores of the original version with a sample from United Kingdom (Evans et al., 2002).

**Figure 1:**
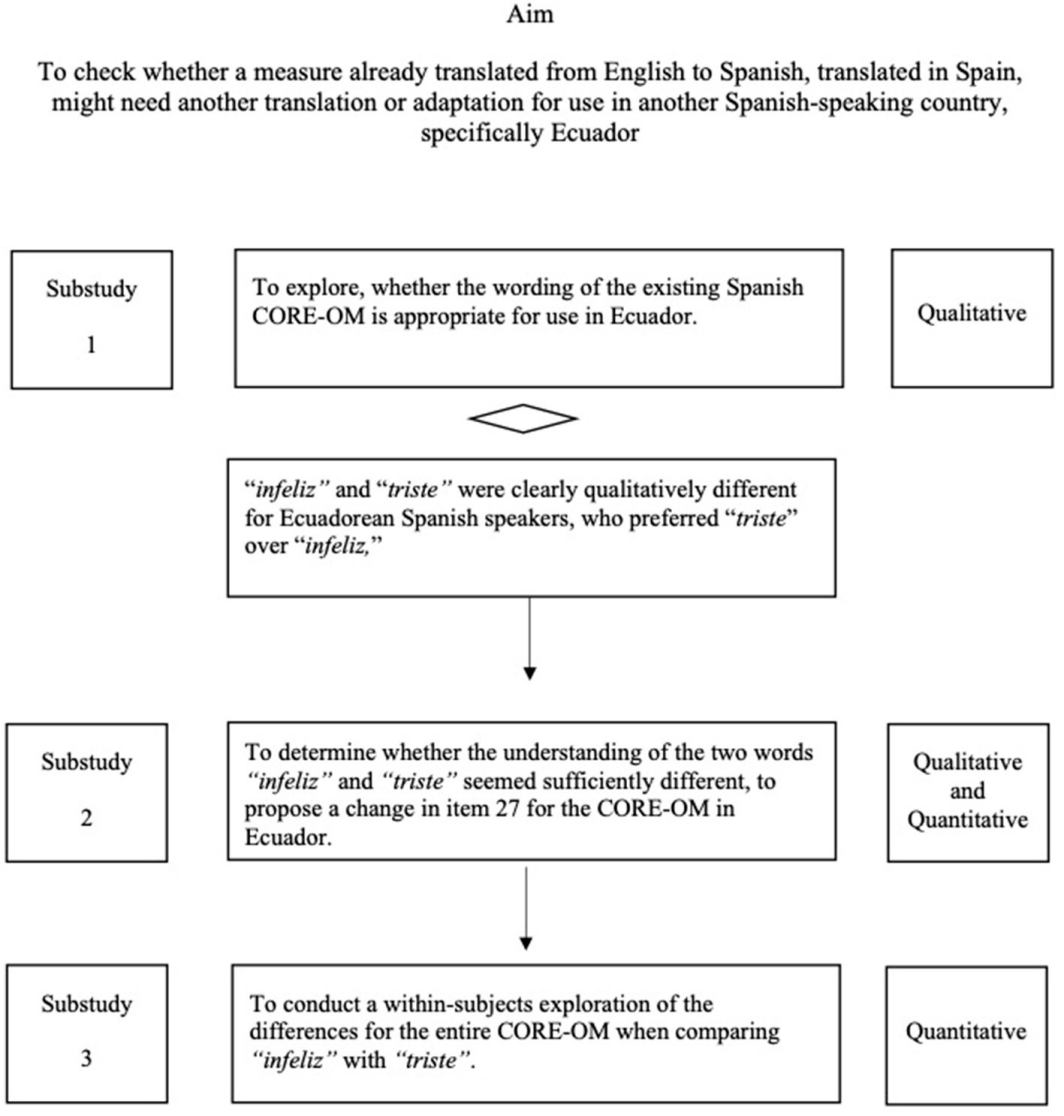
Aim, process, and type of approach of each substudy.

Conventional cross-sectional psychometric analysis of the scores on the 34 items of the CORE-OM (scores from substudy 3 reported here) has shown good psychometric properties concerning acceptability, reliability, and convergent validity in the non-help seeking population in Ecuador (Paz et al., 2020b). This study presents the work done prior to that exploration to make the case for use of the sequential methods reported here, which we believe are new for this task, although widely used individually, for other purposes. Figure 1 shows the process and aims of each substudy, which are presented in detail below.

## Substudy 1

### Rationale

The purpose of the first study was to qualitatively explore whether the wording of the existing Spanish CORE-OM is appropriate for use in Ecuador.

### Methods

This substudy was conducted in two phases. In the first phase, 10 people born in Ecuador, but who had lived in English-speaking countries and are sensitive to cultural differences between those contexts, were asked to translate the CORE-OM from English to Spanish. The participants had a graduate-level education; five of them had degrees in the health sciences (three were clinical psychologists and two had medical degrees) and five had degrees not related to health sciences (one had a business-related degree, one had a degree in social communication, and two had engineering degrees).

These individuals were given the following instructions: “do not translate the items literally and consider each item as a whole. Try to make a translation that can be understood in the Ecuadorian context while conveying the nuances of the English version of each item.” The translations produced were then analyzed to identify consensus and issues for translations for each item. These translations were then compared with the Spanish version of the CORE-OM (Trujillo et al., 2016) to generate a pool of possible translations for each item to be used as alternatives in the second phase of this substudy.

The second phase involved a “think aloud” interview (Charters, 2003) in which participants were asked to talk through their thoughts while completing the measure. This technique was used, because it provides direct information from the possible users and allows the subject to suggest changes or amendments to the questionnaire, including the instructions, the Likert scoring levels, and the items themselves. In this phase, 11 participants were interviewed; none of whom had contributed to the first phase. Their age ranged from 22 to 55 years (mean = 38.4 years, SD = 12.5), six women and five men, nine left formal education after completing high school and two after completing elementary school. We included people from all the main regions in Ecuador (Coast = 2, Galapagos = 2, Highlands = 4, and Amazon = 3), because there are slight variations in the use of Spanish in each region.

After reading each item of the CORE-OM, all the participants were asked the following questions: (1) Do you understand the item? What do you think it means? (2) Do you think the general population could understand this item? If not, why do you think it will be difficult to understand? How would you change the wording to make it more understandable?, and (3) Do you think that a person who is experiencing distress could understand this item? If not, how would you change the wording to make it more understandable?

For items with multiple versions of the translations from phase one, the interviewees were asked which of the multiple versions, such as the one from Spain, would be best understood in Ecuador. All the interviews were audio-recorded and transcribed. The transcriptions were organized by the component of the CORE-OM: instructions, response options, and items. Within each element, information was further organized by the three main questions to the participants: (1) what the participant understood, (2) whether the element is considered comprehensible, and (3) whether the element is appropriate for people experiencing distress. Two independent raters reviewed the transcripts in order to determine whether the participant understood the intended meaning of each item and to collate suggestions for improving the language where needed. The proportion of items rated as intelligible for the different target populations was obtained. For each element of the measure, we defined it as evidence of issues if one or more of these 11 participants considered there was a problem on any of the three questions asked.

### Results

In the first phase, the new forward translations from the 10 people born in Ecuador were robustly similar for most items, and only minimal syntactic variations involving connectors that did not affect meaning were detected. Only for item three the variations in translations were substantial enough to indicate the presence of two different translated versions of the item. The Ecuadorian translations of the items were then compared with the Spanish version, and differences were identified in 20 items. Most differences were simply in word order or, in some cases, in the use of a different tense. For example, for item 9, “I have thought of hurting myself,” for which the Spanish translation is “*He pensado en hacerm daño a m*í *mismo*,” the alternative translation used by three Ecuadorian translators was “*He tenido pensamientos sobre hacerme daño a mí mismo*.” Another example is item 20: “my problems have been impossible to put to one side,” for which the Spanish translation is “*Me ha sido imposible dejar a un lado mis problemas*,” while the alternative translation proposed by six Ecuadorian translators was “*Ha sido imposible poner mis problemas a un lado*.” All the relevant translations for each item, such as the Spanish version, were then used in the second phase of this substudy as alternatives (all translations are available in Supplementary Material 1).

In the second phase of the study, none of the elements reached the predefined level of concern of the authors, i.e., all items were deemed understandable and appropriate for an Ecuadorian general population, and for people experiencing distress, by the people interviewed. There were no differences between the responses given by participants from different regions. However, a lesser concern emerged clearly. When the participants were asked which version of each item would be more understandable for the general Ecuadorian population, the version developed in Spain was preferred over the alternative versions in most of the cases (at least 8 of the 11 participants choosing that version). In contrast, for item 27 (English version: “I have felt unhappy,” Spanish version: “*Me he sentido infeliz*”) all the 11 interviewees preferred the translation developed by Ecuadorian translators (“*Me he sentido triste*”). Explanations for this unanimous preference varied a little; some said that “*triste*” is a word that is more commonly used in Ecuador than “*infeliz*.” However, the other participants said that the meaning of the words differs in that “*infeliz*” is something permanent, a personal trait, while “*triste*” refers to an emotion that appears only when something bad happens: “when I think about a person that is unhappy[“*infeliz*”], I think that person has never been happy; however when I think in somebody who is sad [“*triste*”], I am thinking in a person that have a bad day or had something bad happen to them, and s|he is feeling sad in that moment.” Participant responses for each preferred item are included in the Supplemental Material 1.

The qualitative information provided by this substudy indicated that almost all the elements (instructions, scoring format, and items) of the Spanish translation of the CORE-OM are understandable and appropriate to be used in Ecuador. The one emerging issue was with item 27, for which the Ecuadorian translation was preferred over the original Spanish, highlighting a perceived difference in meanings between “*infeliz*” and “*triste*.” In light of this, substudy 2 was designed to quantify this difference by analyzing the ratings of people to these and related adjectives.

## Substudy 2

### Rationale

Substudy 1 had shown that “*infeliz*” and “*triste*” were clearly qualitatively different for Ecuadorian Spanish speakers, who preferred “*triste*” over “*infeliz*,” which was the word in the existing Spanish CORE-OM. The objective of substudy 2 was to determine whether the understanding of the two words seemed sufficiently quantitatively different, potentially enough to propose a change in item 27 for the CORE-OM in Ecuador. We constructed a questionnaire to quantify possible differences between the words. The substudy considered responses of individuals to that questionnaire, and then a focus group was used to collect further qualitative data about the differences in meanings between “*infeliz*” and “*triste*” and reasons to choose one option over the other for the Ecuadorian general and help-seeking populations.

### Methods

#### Participants

In this substudy, there were 54 participants, all psychology students (35 females) with age ranging from 18 to 22 ($\bar{x}$ = 18.41 years, *SD* = 0.88). We approached two different university classes and asked students to participate in the study; participation was voluntary and no extra credit was given.

#### Procedures and Instruments

A short questionnaire with three sections was specifically designed for this part of the substudy. The questionnaire with the English translation can be found in Supplementary Material 2. In each of these sections, five items were presented to the participants. These included the Spanish versions for item 27: “*Me he sentido infeliz*” and the Ecuadorian alternative “*Me he sentido triste*,” with three other options “*Me he sentido intimidado/a*” [I have felt afraid], “*Me he sentido tenso/a*” [I have felt stressed], and “*Me he sentido desesperanzado/a*” [I have felt hopeless]. The inclusion of the three variant items was designed to give context to differences found between them.

In the first section, the participants answered for each item how they have felt today and how they have felt in the last 7 days using a visual analog scale (VAS). A pole of the scale indicated “this does not fit how I have felt at all today/in the last 7 days,” while the other side indicates “this really fits how I have felt at times/much/all of today/in the last 7 days.” The focus was the paired difference ratings of “*triste*” and “*infeliz*” in location on that VAS. We computed the mean differences and Cohen's *d* values for effect sizes (Cohen's *d1* and *dz* for paired data; *d1* compares the mean paired difference with the standard deviation of the baseline value, and *dz* compares that mean with the standard deviation of the difference). All three were reported together with their bootstrapped 95% confidence intervals (CI).

The second section of the questionnaire was designed to capture how people perceived each item and whether that could be distressing or not. Again, answers were on a VAS from “not at all/highly unlikely” to “could quite seriously disturb people,” and the exploration was of the paired difference in location for the two adjectives. In this section, the participants were asked to answer the following questions:

1. How much do you think this item might upset some people?
2. Do you think this item might put someone off answering a questionnaire if they found it in there?
3. Do you think some people might not answer this item honestly?

The third and last section was designed as a different quantitative exploration of differences between “*infeliz*” and “*triste*” by mapping them to other words. In this section, students were asked to consider a list of adjectives: “*culpable*” [guilty], “*asqueado*” [nauseous], “*avergonzado*” [ashamed], “*deprimido*” [depressed], “*enfadado*” [angry], “*irritable*” [irritable], “*desesperado*” [desperate], “*miserable*” [miserable], “*desdichado*” [unfortunate], and for each of those to say which they thought was closest to each of the five adjectives included in the items presented in previous sections (“*infeliz*,” “*triste*,” “*intimidado/a*,” “*tenso/a*,” and “*desesperanzado/a*”).

The difference in paired proportions of association of each of the five adjectives with (“*infeliz*” and “*triste*”) was tested by McNemar's test of paired association. As there were five comparisons to reduce the risk of overemphasizing differences, it was agreed *a priori* to report both association differences smaller than *p* = 0.05 and smaller than 0.01 (the Bonferroni correction). All the statistical analyses performed for this substudy were conducted using the R software (R Core Team, 2020).

The final component of the substudy was the focus group designed to check if there were any other issues about the possible differences between the two adjectives. The focus group was convened once the students completed the questionnaire, and the students were invited to talk about of the items presented in the questionnaire. Questions included in the conversation were:

1. How would you describe a person that is… (completed with each one of the five items)?
2. How can we differentiate between a person that is … and a person that is…? (pairs of the five items were considered for this question),
3. If these items were included in a questionnaire to be used with general Ecuadorian population, could there be misunderstanding between the items?
4. How do they have to be written to avoid misunderstandings?

The strategy of the focus group moderator was to look for consensus with particular reference to the two words “*infeliz*” and “*triste*” and to discuss possible new meanings. The focus groups were audio-recorded, and verbatim transcriptions were created. The analysis consisted of a minimal thematic one to identify the consensus in meanings and to check for new words that might be better than “*infeliz*” and “*triste*” for item 27.

### Results

Table 1 shows means, standard deviations of each item, and the difference and CIs of the items “today” and “last 7 days”. The effect sizes were small and CIs for the differences were wide; however, they did not include zero, showing that the differences between “*infeliz*” and “*triste*” were unlikely to have arisen by chance.

**Table 1:** Mean, standard deviations, difference, confidence intervals, and effect size between “*infeliz*” and “*triste*” for today and last 7 days (substudy 2).

	***“Infeliz”***	***“Triste”***	**MeanDifference**	**Cohen *d1^***b***^***	**Cohen *dz^***c***^***
	***M* (*SD*)**	***M* (*SD*)**	**[95% CI] ^**a**^**	**[95% CI] ^**a**^**	**[95% CI] ^**a**^**
Today	18.9 (23.3)	25.7 (29.8)	−6.8 [−13.4, −1.0]	−0.29 [−0.69, −0.03]	−0.29 [−0.56, −0.04]
Last 7 days	28.4 (28.8)	34.3 (31.7)	−6.0 [−12.1, −0.6]	−0.21 [−0.48, 0]	−0.27 [−0.51, −0.01]

a *95% Bootstrapped confidence interval.*

b *Cohen's d1 compares the mean paired difference with the standard deviation of the baseline.*

c *Cohen's dz compares the mean paired difference with the standard deviation of the difference*.

Table 2 shows means, standard deviations of each item, and the difference and CIs for the three studied variables (upset some people, stop people from answering, and prevent answering honestly). CIs were wide, but all included zero, indicating that there was no difference between the ratings for “*triste*” and “*infeliz*.”

**Table 2:** Paired differences between “*infeliz*” and “*triste*” for ratings (substudy 2).

	***“Infeliz”***	**“*Triste”***	**Mean difference**	**Cohen *d1***	**Cohen *dz***
	***M* (SD)**	***M* (SD)**	**[95% CI] ^**a**^**	**[95% CI] ^**a**^**	**[95% CI] ^**a**^**
Upset some people	42.6 (23.3)	43.6 (25.1)	−1.0 [−7.5, 5.8]	−0.04 [−0.31, 0.24]	−0.04 [−0.31, 0.23]
Stop people from answering	44.6 (28.8)	42.8(25.3)	1.9 [−4.9, 8.3]	−0.04 [−0.31, 0.24]	−0.04 [−0.31, 0.23]
Prevent answering honestly	59.2 (29.5)	59.6(27.5)	−0.4 [−7.9, 7.0]	−0.04 [−0.31, 0.24]	−0.04 [−0.31, 0.23]

a *95% Bootstrapped confidence interval.*

b *Cohen's d1 compares the mean paired difference with the standard deviation of the baseline.*

c *Cohen's dz compares the mean paired difference with the standard deviation of the difference*.

In the third section, where the participants were asked to tick the adjective that presents the closest meaning for each item, the words with closest meaning to “*infeliz*” were “*miserable*,” “*deprimido*,” and “*desdichado*,” while those for “*triste*” were “*deprimido*” and “*desdichado*” (matching). McNemar's test showed that the differences between “*triste*” and “*infeliz*” were statistically significant at *p*< *0.05* against “*deprimido*” *(*χ^2^ = 5.8, *p* = 0.016) and against “*miserable*” (χ^2^ = 10.24, *p* = 0.0014). Applying a pre-planned Bonferroni correction for having five tests, i.e., testing against *p* < 0.01, it is clear that the greater association of “*miserable*” with “*infeliz*” rather than with “*triste*” remains statistically significant.

Findings from the focus group reveal that most of the participants believe that there are several differences between the two words. In general, the participants thought that “*infeliz*” is a “kind of a lifestyle,” “it is a more permanent and global feeling,” and “something that is catastrophic.” In the case of “*triste*,” the participants believe that this is a “mood state that the person experiences,” “it is a unique emotion,” “a momentary distress produced by a specific event.” Most of the participants (40 of 54) indicated that “*triste*” will be a better option for a questionnaire assessing psychological distress, because “‘*triste*' is a most commonly used word than ‘*infeliz*' for people in Ecuador,” “the meaning of ‘*triste*' is more direct than ‘*infeliz*',” and “‘*infeliz*' is an insult, its use depends on the context.” The participants did not propose any new word that can be used instead of “*triste*” or “*infeliz*.”

This substudy confirmed both qualitatively in the focus group and quantitatively what we found in substudy 1. In Ecuador, the words “*infeliz*” and “*triste*” are perceived differently. Specifically, we found that these words are perceived differently in word pairings and when presented in items for self-rating, as well as in open group discussion (i.e., focus group). However, the results did not show differences in participant ratings of whether the words would upset some people, stop people from answering, and prevent answering honestly. The effect size of the difference in mean self-ratings between the two words was small, but it suggested that a choice is needed to be made whether or not to have a new Spanish CORE-OM for Ecuador that is different from the translation created in Spain on just one item. However, it was still not clear that changing “*triste*” for “*infeliz*” in the CORE-OM would change the psychometric properties of the questionnaire enough to justify the creation of a new version.

## Substudy 3

### Rationale

Substudy 3 was a formal, within-subjects exploration of the differences for the entire CORE-OM when comparing “*triste*” with “*infeliz*.” The study was embedded in a traditional psychometric exploration of the 34 items of the original Spanish CORE-OM (Trujillo et al., 2016). The analysis contextualizes the effect of the word change against two between groups effects: gender, and help-seeking versus non-help-seeking status. Gender is often an important issue to consider in mental health that frequently marks a difference in scores, although likely smaller than the help-seeking versus non-help-seeking difference. Statistical analysis involved both within participants effect of word (“*infeliz*” versus “*triste*”) and comparing those with between group effects (i.e., gender, help-seeking versus non-help-seeking). To avoid the word effect being inseparable from order of item presentation, we included a third between groups effect of order of comparison (“*infeliz*” as item 27 and “*triste*” as item 35 vs. those who saw “*triste*” as item 27 and “*infeliz*” as item 35).

### Methods

#### Participants

In total, 1,233 participants were invited to complete the variation of the CORE-OM questionnaire with 35 items, i.e., with the additional item “*Me he sentido triste*.” The participants were from two different populations: help-seeking and non-help-seeking. The help-seeking sample included clients asking for psychotherapy treatment in two centers. These centers offer psychological treatment for symptoms associated with depression and anxiety, and adaptation issues. In total, 171 clients completed the questionnaire before starting psychological treatment, and there were no missing item data. That subsample consisted of 96 females, 74 males (and one person who did not declare a gender), and they had a mean age of 29.16 years (*SD* = 9.43; range = 18–58). The non-help-seeking sample included 632 students of a private university and 429 non-student participants, but 38 student and 21 non-student participants did not complete all items of the CORE-OM with 35 items and were omitted from the analysis. Of the remaining participants in this non-help-seeking sample, 556 were females, 443 were males, and three persons did not declare their gender. Their mean age was 28.28 years (*SD* = 11.56, age range = 18–80). All the participants who completed the questionnaire were living in Quito, the capital of Ecuador, when they completed the questionnaire. More details about the non-help-seeking sample can be found in Paz et al. (2020b), which reports a traditional psychometric analysis of data using only “*infeliz*”: i.e., analysis of the existing Spanish translation.

#### Procedures and Instruments

We created two versions of the questionnaire, each with 35 items instead of the usual 34. The first version contains all 34 items of the Spanish version of CORE-OM (Trujillo et al., 2016), such as “*Me he sentido infeliz*” in its usual position as item 27 but with an extra item (item 35: “*Me he sentido triste*”). In the second version, the order of the two items was reversed: item 27 was “*Me he sentido triste*” and item 35 was “*Me he sentido infeliz*.” The two versions of the questionnaire were randomly allocated to participants in both the help-seeking and non-help-seeking samples.

#### Data Analysis

This started with a full analysis of variance (ANOVA) with predictor variables being the three between groups effects (gender, help seeking versus non-help-seeking, and item order) and the predictor of primary interest: within-participant word effect, i.e., “*triste*” versus “*infeliz*,” with all interactions. The dependent variable was the item score for the item, i.e., “*Me he sentido infeliz*” or “*Me he sentido triste*.” This ANOVA tests the null hypotheses of no effect on mean score of the between-group variables and the within-participant effect of language, and it tests for all possible interactions between the predictors. Although the statistical significance of all effects and interactions was of interest, the main focus was on the effect of language, i.e., of the mean difference in scores between the two words “*infeliz*” and “*triste*” and particularly on how this effect compared with the mean difference for the other effects (gender and help-seeking). Including the order of presentation of the two words allowed any effect of word to be separated from any effect of order, in particular that one of the two versions became the last item in the measure. After analyzing item scores, we compared mean domain and total scores of the CORE-OM with either “*infeliz*” or “*triste*” to determine the impact on scores that might be used clinically or in research. In addition to those means, we tested how the internal consistency of scores changed between a version with “*infeliz*” and one with “*triste*,” reporting this with the bootstrapped 95% confidence interval. Statistical analysis were performed using the R software (R Core Team, 2020). For the ANOVA, the package rstatix was used (Kassambara, 2021).

### Results

**Figure 2:**
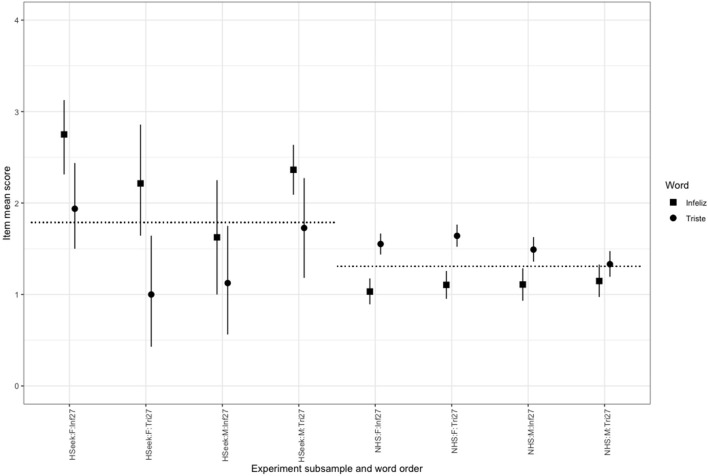
Mean item score by word, sample, gender, and item order. Points mark item mean scores by group, vertical error bars are bootstrapped 95% confidence intervals. Horizontal reference lines are aggregated means for the help-seeking and non-help-seeking samples. Code of subsamples is Sample:Gender:Position specifically: HSeek, help-seeking; NHS, non-help-seeking; Inf27, “*Infeliz*” in item 27; Tri27, “*Triste*” in item 27. Y axis scaled from 0 to 4: the full range of the item.

The full ANOVA table is shown in Table 3. It can be seen from the ANOVA table that the effect of sample, i.e., help-seeking versus non-help-seeking, is highly significant [*F*_(1, 2, 308)_ = 248, *p* < 0.001] as one would expect. This strong effect is reflected in a mean difference between the groups, for the mean of the “*infeliz*” and the “*triste*” responses, of 1.06 (2.36 versus 1.3 on a scale of 0–4). By contrast, the simple effects of gender (mean difference 0.07), word (mean difference 0.04, “*infeliz*” minus “*triste*”) and position (i.e., whether “*infeliz*” was in its usual position as item 27 or as item 35; mean difference 0.02, higher if “*infeliz*” was item27) were non-significant. However, interpretation is complicated as two of the interactions containing the word effect were statistically significant. As can be seen from the table, those were the three-way interaction of gender, position, and word [*F*_(1, 2, 308)_ = 7,296, *p* = 0.007], and the two-way interaction of position and word [*F*_(1, 2, 308)_ = 91.56, *p* < 0.001]. Removing the four-way interaction and the non-significant three-way interactions did not change the significance of any of the other effects. In order to understand these findings in terms of the means, we plotted these (Figure 2) with 95% confidence intervals to give a clear decomposition of the effects.

**Table 3:** ANOVA table showing full analysis of variance of item score (substudy 3).

**Effect**	***df*_**Num**_**	***df*_**Den**_**	***F***	***p***	***ges***
Gender	1	2308	1.934	0.16	0.00083
Sample	1	2308	248	<0.001^**^	0.097
Position	1	2308	0.036	0.85	0.000016
Words	1	2308	0.665	0.42	0.00029
Gender:sample	1	2308	0.049	0.83	0.000021
Gender:position	1	2308	0.044	0.83	0.000019
Sample:position	1	2308	0.252	0.61	0.00011
Gender:words	1	2308	0.991	0.32	0.00043
Sample:words	1	2308	0.145	0.70	0.000062
Position:words	1	2308	91.56	<0.001^**^	0.038
Gender:sample:position	1	2308	9.652	0.002^*^	0.004
Gender:sample:words	1	2308	0.019	0.89	0.0000083
Gender:position:words	1	2308	7.296	0.007^*^	0.003
Sample:position:words	1	2308	3.051	0.08	0.001
Gender:sample:position:words	1	2308	0.129	0.72	0.000056

*df_Num_, degrees of freedom numerator; df_Den_, degrees of freedom denominator; ges, generalized eta squared measure of effect size.*

* *p < 0.05,*

** *p < 0.001*.

The primary interest was in the effect sizes. The means are shown in Figure 2 with circles for “*triste*” whether that came as item 27 or item 35, and squares for “*infeliz*.” The vertical lines mark the bootstrapped 95% confidence intervals, and the horizontal reference lines are for the mean across both words, by sample: help-seeking or non-help-seeking. The four sets of values within the two samples separated by gender and by the order in which the word was first seen by the participant so HSeek:F:Inf27 indicates that the mean is for the women in the clinical sample who saw the wording with “*infeliz*” as item 27 and NHS:M:Tri27 indicates the mean for the men in the non-help-seeking sample who saw the word “*triste*” as item 27. The data for each word have been plotted nudged slightly on the x axis to prevent overprinting.

The vertical distance between the aggregate mean for help-seeking and non-help-seeking samples in Figure 2 shows that both versions of the item, “*triste*” or the original translation “*infeliz*,” differentiate clearly between those two groups. It can also be seen that the acknowledgment for “*triste*” is always stronger than for “*infeliz*” but with a visible interaction with gender and order of the two wordings of the item with the interaction mainly in the help-seeking sample. Surprisingly, the interaction is strong enough to essentially remove any statistically significant mean difference between help-seeking and non-help-seeking mean scores when “*triste*” is used, not “*infeliz*,” although this is only true for women with “*triste*” as item 27 and for men with “*triste*” as item 35. This is discussed below. These findings clearly confirmed the differences that had emerged through the small sample substudies (1 and 2) but left us with the key questions of how much difference changing “*infeliz*” for “*triste*” would make to the scores of the CORE-OM. Item 27 contributes to three possible scores out of the CORE-OM: the problems domain score (12 items), the non-risk score (28 items), and the total score across all the 34 items.

The mean score across the whole sample for item “*Me he sentido infeliz*” was 1.24 with SD 1.31, and for “*Me he sentido triste*” the score was 1.69 with SD 1.31, a difference of 0.45 indicating that people clearly endorsed “*triste*” more often than “*infeliz*.” Cohen's *d1* was 0.35 with 95% CI from 0.29 to 0.42 and *dz* 0.36, 95% CI 0.29–0.44. That is a substantial difference at the item level. However, the effect on the mean for the problems score was a change from 1.24 to 1.27 (difference = 0.036, 95 % CI [0.031–0.042]); for the mean non-risk score the change is from 1.16 to 1.18 (difference = 0.016, 95 % CI [0.014–0.019]), and finally for the total CORE-OM score, the effect is to change the mean from 1.02 to 1.03 (difference = 0.014, 95 % CI [0.011–0.016]). It can be seen that the impact on any of the scores is very minor.

The final analysis was of the impact on internal reliability. Table 4 shows Cronbach's alpha for each of the three scores when the original item 27 “*Me he sentido infeliz*” is used and again with “*Me he sentido triste*.” Alpha is reported with the 95% bootstrapped CIs as is the difference in alpha with its CI. It can be seen that the effect of the change of word is statistically significant as the CIs do not embrace zero, but the effects are only at the second or third decimal place.

**Table 4:** Cronbach's alpha for each item and the difference between them in relation to the total score, problem item score, and non-risk item score.

	***“Infeliz”***	***“Triste”***	**Difference**
	**Cronbach's alpha [95% CI] ^**a**^**	**Cronbach's alpha [95%CI] ^**a**^**	**Cronbach's alpha [95%CI] ^**a**^**
Total score	0.94 [0.93, 0.94]	0.94 [0.93, 0.94]	−0.002 [−0.003, −0.002]
Problems items score	0.88 [0.87, 0.89]	0.89 [0.88, 0.90]	−0.011 [−0.01, −0.008]
Non-risk items score	0.93 [0.92, 0.94]	0.93 [0.93, 0.94]	−0.003 [−0.004, −0.002]

a * 95% Bootstrapped confidence interval*.

## Discussion

We divide the discussion of this study into two sections: (1) reviewing the stepwise and mixed (qualitative and quantitative) methods we used to explore local language issues and the findings for the use of the existing Spanish translation of the CORE-OM in Ecuador and (2) noting general implications for prevailing methods and myths about using cross-cultural psychometrics of self-report measures.

### The Methodology and Specific Findings

The traditional method to explore possible issues with the Spanish CORE-OM in another country with possible language variation issues would be to give the existing Spanish translation of the CORE-OM to a large sample of Ecuadorians and subject their item scores to psychometric inspection measurement invariance informally by comparing with the findings of Trujillo et al. (2016). We considered this but wanted to identify probable issues, so that any large study would be able to contain alternative wordings if preliminary suggested this might be necessary. We understood that there can be no simple rule to decide when statistically significant differences in item scoring between samples would be sufficient to justify a new version of the measure but started with a clear preference not to create a new, slightly changed translation unless it seemed that not to do would create more issues with comparability than would be created by having different words across two measures.

We began in substudy 1 with a new translation of the measure from the original English to Spanish by Ecuadorian Spanish speakers and then a careful qualitative review of any language and cultural issues arising from that. That showed unanimous agreement that there was a problem with the word “*infeliz*” in one item and that the alternative word “*triste*” was preferred in Ecuador. Although that finding was unanimous, it gave no indication of the quantitative effect changing the word would have, so we conducted a second small sample exploration, this time using both quantitative and qualitative methods. The Participants were first asked to self-rate several items on a VAS, such as “*Me he sentido triste*” and “*Me he sentido infeliz*” for two-time intervals (today and last 7 days). This was done to capture a trait/state distinction, between “*infeliz*” and “*triste*,” respectively, that the participants reported in substudy 1. We also designed some items to detect whether the participants would expect negative reactions to the items in each of its form, and to map words based on perceived semantic grouping. All these questions were developed to quantify the similarity between these two words and the impact that they might have on item functioning. The sample in this second substudy was small (*n* = 54), but the within-participant design gives enough power to detect probable population differences and to give some precision of estimation of the quantitative differences between the two words. This substudy confirmed that the differences were small but not trivial and might impact on scores using the CORE-OM. It was clearly both necessary and ethically justifiable to include both versions of the item in a large *n* study for comparison in substudy 3.

The findings of this last step showed that the change of word produced a statistically significant mean shift, but this was small and complicated by statistically significant interactions with gender and with the order of presentation of the two items. Although difficult to interpret, we believe the interesting interactions represent the combination of an order/recency effect with the state/trait connotations of “*triste*” and “*infeliz*” (respectively) and a gendered sensitivity to trait judgements. At least, this is the only explanation we have for the clear finding that looks strong enough to be replicable. We considered this in relation to cultural issues, and there is some literature on gendered cultural differences, e.g., the Latin American “*macho*” stereotype. We believe the stereotype hides complex gender constructions that are not unique to Latin America and which merit much more empirical exploration than they have had. We are reluctant to jump to the idea that this may be a cultural, geographically restricted finding. We will seek to collaborate with colleagues in other Spanish-speaking Latin American countries and in Spain to see if the effects are robust and to, perhaps, reopen questions of item order, trait/state connotations of mental health measurement, and of gender.

Additionally, we found other interesting results not related to item wording. We found statistically significant interactions of order of presentation of the two words with gender, sample (help-seeking versus non-help-seeking). Of course, rare, random events happen (Hand, 2015), and this may be a chance finding. However, it may be a replicable finding reminding us of the complexity of real-world item responses: responses may vary with order of items, with gender, as well as with word change. It may be that the decision to keep the usual position of the item (item 27 of 34) and to add the alternative wording at the end (as item 35) may have made for a stronger order effect than we might have seen had we randomly shuffled the position of the two items within the 35. However, that would have lost some comparability with the routine presentation of the CORE-OM and was simply not feasible in this study, as we were using the measures on paper.

Whatever the nature of these complex effects, reassuringly, the findings showed that the impacts on domain scores and on internal consistency, although statistically significant, were tiny. Though significant interactions may be fortuitous and may not replicate, that they show that using “*triste*” instead of “*infeliz*” removed any significant mean help-seeking versus non-help-seeking mean difference for particular combinations of word order and gender reinforced our decision not to change the wording for Ecuador.

To summarize, these results show differences in measurement for a within language, between countries use of the Spanish CORE-OM. Reviewing the findings, we felt that the economy and comparability issues of staying with the existing Spanish translation outweighed the tiny changes in scoring, changes that would be entirely subsumed into the development of local referential data (Paz et al., 2020b). We hope to collaborate with other researchers in other Spanish-speaking Latin American countries to explore this further and believe that this one-word Spanish–Spanish example of a within-language difference is probably at the lower end of such issues, certainly smaller than the challenges translating across languages when, of course, none of the original words can be retained.

### General Implications

Despite this specific focus, we saw this study as of more general relevance to transcultural use of measures, and to methodology recommending that qualitative exploration precedes quantitative psychometric testing.

An early example of the inefficiency of omitting the careful qualitative exploration of a translation is El-Rufaie and Absood (1987) who translated the Hospital Anxiety and Depression Scales (HADS; Zigmond and Snaith, 1983) from English to Arabic and then got an independent backtranslation that matched the original English well. The item-scale correlations showed 13 items correlating as expected, but the item “I get a sort of frightened feeling like 'butterflies' in the stomach” did not come out as it should have. It seems it was only then that the authors recognized that the phrase “like 'butterflies' in the stomach,” while widely used for anxiety in UK English, had no such associations in Arabic in Saudi Arabia (in most of mainland Europe the term is more associated with being in love than with anxiety). This is the perfect example of the problem with inefficient and/or incomplete pre-testing efforts and jumping prematurely to a large-scale psychometric exploration.

Since 1987, there has been a steady stream of translation guidelines that improve on that used in that study. However, each guideline seems to imply that measurement invariance might be achievable across languages (Epstein et al., 2015b) and sometimes suggest that a particular guideline is better than all the others and the route to a perfect translation. Largely independently of that literature on processes of translation, there is an enormous number of studies exploring “measurement invariance” of questionnaires (Nuevo et al., 2009; Romppel et al., 2017; Scholten et al., 2017; Zanon et al., 2020). Such psychometric explorations of measurement invariance require large samples and often find violations of invariance and recommend new scoring systems for measures, breaking comparability with existing study with the measure. Such reports almost never report the effect size of changed scoring to see whether the difference is important, nor do they show scattergrams and the correlation between the new and old scorings.

The finding of a particular failure of measurement invariance in reliability is congruent with the diverse literature on “reliability generalization” within and between languages, which shows that measurement invariance, at the level of statistically significant differences in internal reliability, is not the norm but the exception, a finding recognized in the APA requirement (Appelbaum et al., 2018) that all studies using multi-item measures must report their own internal reliability and not just cite early studies as if reliability were a fixed property of the measure. This will expose where psychometric properties are clearly hopeless, e.g., internal reliability of −0.01 to 0.3 for the scales of the 26-item Eating Attitudes Test in English completed by bilingual Nigerians (Evans et al., 1997). This can also raise cautionary notes where reliability may be usable but clearly different from that found in other samples.

A more recent example is Giusti et al. (2019) who explored the HADS in Dutch in people with chronic pain. Detailed analyses of large samples with both CFA and IRT found failure of measurement invariance, and the authors proposed for the use with this population a scoring that only considered 11 of the 14 items of the HADS and rescaled one item to be scored 2-1-1-0 rather than 3-2-1-0. The study is firm that this will improve studies using the measure (while acknowledging that all comparison with existing results using the HADS will be lost). No direct comparison with the original scoring is given, but one table allows the scoring to be mapped, one score level to the other (for the 11 items) and that shows a correlation of 0.99 between the two scores. That is not a correlation across the possible scores on the retained items, not a correlation with the original scoring of all 14 items; however, it suggests that the hugely impressive-looking analysis has prioritized some rather elusive invariance over utility and taken large samples and much data analysis to achieve this.

We believe that funders, researchers, and journal editors should be leading a move away from the arid pursuit of measurement invariance across languages and should recognize that small *n* mixed method studies can be used to identify and quantify non-invariance, i.e., the ubiquitous and sometimes complex variance across language variants. We can then recognize that non-invariance may show complex interactions, e.g., of wording by gender, order of presentation, and help-seeking/non-help-seeking status. Such small sample exploration can then indicate whether larger samples are needed for formal psychometric exploration (as in our substudy 3).

Many existing psychometric explorations of short, wide coverage outcome measures suitable for routine use in psychotherapies show neither clean factor structures nor unidimensionality of scores (Fried et al., 2016). These are not perfect rulers, weighing scales, or blood tests, and differences in measurement properties can be detected even in the same language.

These ideas are not completely new: a qualitative dimension to test translation is now included in most translation guidelines. International Test Commission (2001), for example, divide the test adaptation process into several stages, and the initial ones include qualitative explorations of the construct relevance between culture, as well as possible explorations of the semantic equivalence of items by engaging the target audience through methods such as cognitive interviewing. However, the authors of these guidelines refer to cultural differences, which they consider irrelevant to the use of the test as “nuisance variables” that need to be minimized, yet it is not clear how we can assess how a test actually functions in a new setting without fully understanding those cultural differences in the first place. It seems that lip service only is paid to the differences in meaning-making between cultures, while huge energy is spent on deploying sophisticated statistical methodologies to find or reject measurement invariance.

### Limitations

There are clear cautions to consider when interpreting these findings. In substudy 1, we tried to interview persons from different regions in Ecuador to capture the differences in the use of language of each region. However, most of the participants had secondary education; so, probably the inclusion of more participants with only primary education would improve generalizability. In substudy 2, all the participants were students, so a similar caution about generalizability should be considered. In substudy 3, more nearly balanced sample sizes would have led to more precise results but seems unlikely to have changed them.

## Conclusion and Recommendations

Self-reported measures are not like blood tests (Paz et al., 2020a). How users answer these measures involves individual and social contexts in which linguistics can be important. Empirical explorations, qualitative and quantitative, such as those reported here, provide empirical information about the specific characteristics of the measure within the population in which it is going to be used. These methods accept that there are no perfect translations or adaptations but such explorations, and transparency in reporting problematic issues, have crucial implications for measure use and for interpretation of scores. Whether the item “I have felt unhappy” from the CORE-OM be translated from English to Spanish as “*Me he sentido infeliz*” or as “*Me he sentido triste*” was the emergent question and practical focus of this study, but the issues are entirely general when translating a self-report questionnaire to another language or even within a language when moving from one context to another. In this study, we found difficulties in the use of a word in just one item, but it can be the case that in other cultures or with other measures, the number of items presents difficulties that can be greater and might compromise the adequate use of the measure, so, what we present is just an example of how to deal with this issue in order to avoid future misuse of the measures in a different culture.

One element of the methods that should be noted is that they emphasize a user-based approach instead of the commonly used expert-based approach (Prakash et al., 2019). This change helps ensure that the findings should generalize to the wide populations, such as people with only moderate education and literacy, with whom these measures are widely used. They may use a language differently from a very well educated group of experts.

Table 5 distills recommendations arising from the findings of this study for translation and for within-language adaptation of psychotherapy measures. Translation is necessary to achieve any usability of a measure coming from one language into another. The gain is that translating a measure may be much cheaper than developing an entirely new measure on in the target language. However, the danger is that it conveys a false sense of equivalence of scores, and that it may miss the existence of such large language or, more probably, cultural differences that it may be inappropriate to translate and better to start fresh in the target language culture. Adaptation addresses the issue that probably most potential users in the target population may indeed understand the original version but may, at the same time, find the language or the culture expectations so alien that scores might have no strong relationships to scores from people answering the measure in the original, host, language, and culture. The gain of a good adaptation is to expose where scores may not be equivalent despite apparently perfect acceptability, the danger is that pride in identity differences and cultural and political differences may produce variants of measures that are unnecessarily different or of no empirically clear comparability at all. For both procedures, we emphasize that user-based methods should be preferred over expert-driven methods in both the translation and adaptation of measures. Both qualitative and empirical quantitative explorations are needed to assure that the items are used adequately in the target population.

**Table 5:** Recommendations for translation and adaptations of outcome measures used in psychotherapy research.

	**Translation between languages**	**Adaptation within languages**
Immediate alternatives we propose	- To prioritize user-based methods (e.g., translators from the target population, use of interviews about the understanding of the measure with the target population)	- To confirm with the target population that the words used by the original version are commonly used an understandable for them. Use interviews or discussion groups with lay people for that purpose (as in substudy 1).
	- To collect different options of the translations, not only from experts in the measured construct, but also with lay people (bilingual) from the target population (as in substudy 1).	- To conduct small quantitative studies to verify the difference in meanings of alternative words or items (as in substudy 2).
	- To conduct small qualitative studies with people of the target population to understand possible variations in the language and the impact that they can have on the understanding of the measure (as in substudy 2).	- To include empirical testing that explore the scores given to the different alternatives of the items and verify whether the scores have enough impact in psychometric properties that justify that they need to be changed (as in substudy 3).
	- To conduct empirical testing of alternative items that emerged in the qualitative exploration (as in substudy 3).	
Research we propose to explore this further	- To compare, empirically, the translations produced by user-based methods (ours) and expert-based methods.	- To replicate our methods (mixed-methods and user-based methods) with other similar measures.
		- To test gender and cultural differences (in the context of origin and in the target context) that can affect the use of the measure.

We hope this study encourages a discussion of pragmatic methods that should be used to address these questions of between language and within language equivalence of self-reported mental health measures usually used in psychotherapy research.

## Data Availability Statement

The original contributions presented in the study are included in the article/Supplementary Material, further inquiries can be directed to the corresponding author.

## Ethics Statement

The studies involving human participants were reviewed and approved by The Institutional Review Board of the Universidad San Francisco de Quito, Ecuador (ref. 2017-113E). The patients/participants provided their written informed consent to participate in this study.

## Author Contributions

CE conceptualized the study conducted for this article. CE and CP designed the substudies. CP and GM conducted data collection. Data analysis and interpretation were conducted by CE. CP drafted the article. All authors conducted a critical revision and approved the final version of the article.

## Conflict of Interest

The authors declare that the research was conducted in the absence of any commercial or financial relationships that could be construed as a potential conflict of interest.

## Publisher's Note

All claims expressed in this article are solely those of the authors and do not necessarily represent those of their affiliated organizations, or those of the publisher, the editors and the reviewers. Any product that may be evaluated in this article, or claim that may be made by its manufacturer, is not guaranteed or endorsed by the publisher.
