# Supplementary material for: “Infeliz” or “Triste”: A Paradigm for Mixed Methods Exploration of Outcome Measures Adaptation Across Language Variants
Source: Front Psychol. 2021 Aug 9;12:695893. doi: 10.3389/fpsyg.2021.695893 (PMC8381247; doi:10.3389/fpsyg.2021.695893)
Supplement: Supplementary file 1 [file Table_1.DOCX]

S1. Items that present significant differences when translated by Ecuadorians and the number of participants that select each item a preferred item (substudy 1).

| **Items** | **English version** | **Spanish version** | ***n*** | **Alternative version proposed by Ecuadorian translators** | ***n*** |
| --- | --- | --- | --- | --- | --- |
| 1 | I have felt terrible alone and isolated | Me he sentido muy solo y aislado | 10 | Me he sentido terriblemente solo y aislado | 1 |
| 3 | ‘I have felt I have someone to turn to for support when needed’ | He sentido que tenía alguien en quien apoyarme cuando lo he necesitado | 8 | He sentido que tengo alguien a quien recurrir en busca de apoyo cuando lo necesito | 2 |
|  |  |  |  | He sentido que tenía alguien a quien acudir para recibir apoyo cuando lo necesitaba | 1 |
| 5 | ‘I have felt totally lacking in energy and enthusiasm’ | Me he sentido totalmente sin energía ni entusiasmo | 9 | He sentido una falta total de energía y entusiasmo | 2 |
| 6 | ‘I have been physically violent to others’ | He sido violento físicamente hacia los demás | 9 | He sido físicamente violento con otros. | 2 |
| 7 | ‘I have felt able to cope when things go wrong’ | Me he sentido capaz de afrontar las cosas cuando han ido mal | 9 | He estado en capacidad de enfrentar las cosas cuando han ido mal | 2 |
| 8 | ‘I have been troubled by aches, pains or other physical problems’ | He tenido molestias, dolores y otros problemas físicos | 9 | He tenido malestar, dolor o algún otro problema físico | 2 |
| 9 | ‘I have thought of hurting myself’ | He pensado en hacerme daño a mí mismo | 8 | He tenido pensamientos sobre hacerme daño a mí mismo | 3 |
| 10 | ‘Talking to people has felt too much for me’ | Me ha costado mucho hablar con la gente | 8 | Hablar con la gente ha sido demasiado para mí. | 3 |
| 12 | ‘I have been happy with the things I have done’ | Me he sentido satisfecho con las cosas que he hecho | 9 | Me he sentido feliz con las cosas que he hecho | 2 |
| 13 | ‘I have been disturbed by unwanted thoughts and feelings’ | Me han inquietado pensamientos y sentimientos no deseados | 8 | Me han perturbado pensamientos y sentimientos no deseados | 3 |
| 16 | ‘I made plans to end my life’ | He hecho planes para acabar con mi vida | 8 | He hecho planes para terminar con mi vida | 3 |
| 17 | ‘I have felt overwhelmed by my problems’ | Me he sentido agobiado por mis problemas | 9 | Me he sentido abrumado por mis problemas | 2 |
| 18 | ‘I have had difficulty getting to sleep or staying asleep’ | He tenido dificultad para conciliar el sueño o permanecer dormido | 8 | He tenido dificultad para conciliar el sueño o permanecer dormido | 3 |
| 20 | ‘My problems have been impossible to put to one side’ | Me ha sido imposible dejar a un lado mis problemas | 8 | Ha sido imposible poner mis problemas a un lado. | 3 |
| 25 | ‘I have felt criticized by other people’ | Me he sentido criticado por los demás | 8 | Me he sentido criticado por otras personas | 3 |
| 27 | ‘I have felt unhappy’ | Me he sentido infeliz | 0 | Me he sentido triste | 11 |
| 28 | ‘Unwanted images or memories have been distressing me’ | Me han angustiado imágenes o recuerdos no deseados | 8 | Imágenes o memorias no deseadas me han angustiado | 3 |
| 31 | ‘I have felt optimistic about my future’ | Me he sentido optimista sobre mi futuro | 8 | Me he sentido optimista acerca de mi futuro. | 3 |
| 32 | ‘I have achieved the things I wanted to | He conseguido las cosas que quería | 8 | He logrado las cosas que me he propuesto | 3 |
| 34 | ‘I have hurt myself physically or taken dangerous risks with my health’ | Me he hecho daño físicamente o he puesto en peligro mi salud | 8 | Me he lastimado a mí mismo físicamente o he tomado riesgos peligrosos para mi salud | 3 |
