# Supplementary material for: “Infeliz” or “Triste”: A Paradigm for Mixed Methods Exploration of Outcome Measures Adaptation Across Language Variants
Source: Front Psychol. 2021 Aug 9;12:695893. doi: 10.3389/fpsyg.2021.695893 (PMC8381247; doi:10.3389/fpsyg.2021.695893)
Supplement: Supplementary file 2 [file Table_2.DOCX]

S2. The questionnaire used for substudy 2

**CUESTIONARIO DE BIENESTAR PSICOLÓGICO**

*[PSYCHOLOGICAL WELL BEING QUESTIONNAIRE]*

**Edad** *[Age]***: _________________________**

**Género** *[Gender]***: ____Masculino** *[Male]* **____Femenino** *[Female]*

1. Estos son ítems de un cuestionario y queremos comparar las puntuaciones que reciben cuando son respondidos por personas que viven en Ecuador. Lee cada uno de los ítems y piensa en cuanto encaja en **como te has sentido hasta ahora el día de hoy**. Por favor pon un punto en la línea de abajo que va desde *“esto no encaja para nada en como me he sentido el día de hoy”* a *“esto encaja perfectamente en como me he sentido la mayor parte del tiempo el día de hoy” [These items are part of a questionnaire and we want to compare the scores when they are answered by people living in Ecuador. Read each one of the items and think about how it fits in* ***how you have felt so far today****. Please put a point on the bottom line that goes from “This does not fit at all in how I have felt today ” to “This fits perfectly into how I have felt today”]*

Me he sentido irritado, molesto *[I have felt irritated, annoyed]*

| Esto no encaja para nada en como me he sentido el día de hoy  *[This does not fit at all in how I have felt today]* |  |  | Esto encaja perfectamente en como me he sentido la mayor parte del tiempo el día de hoy  *[This fits in perfectly into how I have felt most of the time today]* |
| --- | --- | --- | --- |

Me he sentido infeliz *[I have felt unhappy]*

| Esto no encaja para nada en como me he sentido el día de hoy  *[This does not fit at all in how I have felt today]* |  |  | Esto encaja perfectamente en como me he sentido la mayor parte del tiempo el día de hoy  *[This fits in perfectly into how I have felt most of the time today]* |
| --- | --- | --- | --- |

Me he sentido tenso ansioso o nervioso *[I have felt tense, anxious or nervous]*

| Esto no encaja para nada en como me he sentido el día de hoy  *[This does not fit at all in how I have felt today]* |  |  | Esto encaja perfectamente en como me he sentido la mayor parte del tiempo el día de hoy  *[This fits in perfectly into how I have felt most of the time today]* |
| --- | --- | --- | --- |

Me he sentido triste *[I have felt sad]*

| Esto no encaja para nada en como me he sentido el día de hoy  *[This does not fit at all in how I have felt today]* |  |  | Esto encaja perfectamente en como me he sentido la mayor parte del tiempo el día de hoy  *[This fits in perfectly into how I have felt most of the time today]* |
| --- | --- | --- | --- |

Me he sentido desesperanzado o sin esperanza *[I have felt despairing or hopeless]*

| Esto no encaja para nada en como me he sentido el día de hoy  *[This does not fit at all in how I have felt today]* |  |  | Esto encaja perfectamente en como me he sentido la mayor parte del tiempo el día de hoy  *[This fits in perfectly into how I have felt most of the time today]* |
| --- | --- | --- | --- |

1. Ahora lee cada uno de los ítems y piensa en cuanto encaja en **como te has sentido en los últimos siete días**. Por favor pon un punto en la línea de abajo que va desde *“esto no encaja para nada en como me he sentido en los últimos siete días”* a *“esto encaja perfectamente en como me he sentido en los últimos siete días” [Now read each one of the items and think about how it fits in* ***how you have felt in the last seven days****. Please put a point on the bottom line from “this does not fit at all in how I have felt in the last seven days” to “this fits perfectly into how I have felt most of the last seven days”]*

Me he sentido irritado, molesto *[I have felt irritated, annoyed]*

| *Esto no encaja para nada en como me he sentido en los últimos siete días*  *[This does not fit at all in how I have felt in the last seven days]* |  |  | *Esto encaja perfectamente en como me he sentido en los últimos siete días*  *[This fits perfectly into how I have felt most of the last seven days]* |
| --- | --- | --- | --- |

Me he sentido infeliz *[I have felt unhappy]*

| *Esto no encaja para nada en como me he sentido en los últimos siete días*  *[This does not fit at all in how I have felt in the last seven days]* |  |  | *Esto encaja perfectamente en como me he sentido en los últimos siete días*  *[This fits perfectly into how I have felt most of the last seven days]* |
| --- | --- | --- | --- |

Me he sentido tenso ansioso o nervioso *[I have felt tense, anxious or nervous]*

| *Esto no encaja para nada en como me he sentido en los últimos siete días*  *[This does not fit at all in how I have felt in the last seven days]* |  |  | *Esto encaja perfectamente en como me he sentido en los últimos siete días*  *[This fits perfectly into how I have felt most of the last seven days]* |
| --- | --- | --- | --- |

Me he sentido triste *[I have felt sad]*

| *Esto no encaja para nada en como me he sentido en los últimos siete días*  *[This does not fit at all in how I have felt in the last seven days]* |  |  | *Esto encaja perfectamente en como me he sentido en los últimos siete días*  *[This fits perfectly into how I have felt most of the last seven days]* |
| --- | --- | --- | --- |

Me he sentido desesperanzado o sin esperanza [*I have felt despairing or hopeless]*

| *Esto no encaja para nada en como me he sentido en los últimos siete días*  *[This does not fit at all in how I have felt in the last seven days]* |  |  | *Esto encaja perfectamente en como me he sentido en los últimos siete días*  *[This fits perfectly into how I have felt most of the last seven days]* |
| --- | --- | --- | --- |

1. Por favor, con respecto a cada uno de los ítems mostrados anteriormente, indica con un punto en la línea de abajo la respuesta que consideres más adecuada. *[Please, for each one of the items shown above, indicate with a dot on the line below the answer that you considerer most appropriate.]*

**Ítem 1: Me he sentido irritado, molesto** *[I have felt irritated, annoyed]*

- Pensando en toda la población general del Ecuador: ¿cuánto piensas que este ítem podría perturbar a algunas personas? *[Thinking of the entire general population of Ecuador: ¿ how much you think this item might upset some people? ]*

| Para nada / poco probable |  |  | Podría seriamente perturbar a algunas personas *[It could seriously disturb some people]* |
| --- | --- | --- | --- |

*[Not at all / unlikely]*

- Pensando en toda la población general del Ecuador: ¿piensas que este ítem haría que algunas personas dejen de contestar el cuestionario si lo encontrarán ahí? *[Thinking of the entire general population of Ecuador: ¿* *do you think this item might put someone off answering a questionnaire if they found it in there? ]*

| Para nada / poco probable  *[Not at all / unlikely]* |  |  | Es bastante probable que las personas pueda dejar de contestar al cuetionario *[It is quite probable that people could stop answering the questionnaire]* |
| --- | --- | --- | --- |

- Pensando en toda la población general del Ecuador: ¿piensas que algunas personas podrían no contestar este ítem de manera totalmente honesta? *[Thinking of the entire general population of Ecuador: ¿* *do you think some people might not answer this item honestly? ]*

| Para nada / poco probable  *[Not at all / unlikely]* |  |  | Es bastante probable que las personas no contesten de manera totalemente honesta *[It is quite probable that people will not answer honestly]* |
| --- | --- | --- | --- |

**Ítem 2: Me he sentido infeliz** *[I have felt unhappy]*

- Pensando en toda la población general del Ecuador: ¿cuánto piensas que este ítem podría perturbar a algunas personas? *[Thinking of the entire general population of Ecuador: ¿how much you think this item might upset some people? ]*

| Para nada / poco probable  *[Not at all / unlikely]* |  |  | Podría seriamente perturbar a algunas personas *[It could seriously disturb some people]* |
| --- | --- | --- | --- |

- Pensando en toda la población general del Ecuador: ¿piensas que este ítem haría que algunas personas dejen de contestar el cuestionario si lo encontrarán ahí? *[Thinking of the entire general population of Ecuador: ¿do you think this item might put someone off answering a questionnaire if they found it in there? ]*

| Para nada / poco probable  *[Not at all / unlikely]* |  |  | Es bastante probable que las personas pueda dejar de contestar al cuetionario *[It is quite probable that people could stop answering the questionnaire]* |
| --- | --- | --- | --- |

- Pensando en toda la población general del Ecuador: ¿piensas que algunas personas podrían no contestar este ítem de manera totalmente honesta? *[Thinking of the entire general population of Ecuador: ¿* *do you think some people might not answer this item honestly? ]*

| Para nada / poco probable  *[Not at all / unlikely]* |  |  | Es bastante probable que las personas no contesten de manera totalemente honesta *[It is quite probable that people will not answer honestly]* |
| --- | --- | --- | --- |

**Ítem 3: Me he sentido tenso, ansioso o nervioso** *[I have felt tens anxious or nervous]*

- Pensando en toda la población general del Ecuador: ¿cuánto piensas que este ítem podría perturbar a algunas personas? *[Thinking of the entire general population of Ecuador: ¿how much you think this item might upset some people? ]*

| Para nada / poco probable  *[Not at all / unlikely]* |  |  | Podría seriamente perturbar a algunas personas *[It could seriously disturb some people]* |
| --- | --- | --- | --- |

- Pensando en toda la población general del Ecuador: ¿piensas que este ítem haría que algunas personas dejen de contestar el cuestionario si lo encontrarán ahí? *[Thinking of the entire general population of Ecuador: ¿do you think this item might put someone off answering a questionnaire if they found it in there?]*

| Para nada / poco probable  *[Not at all / unlikely]* |  |  | Es bastante probable que las personas pueda dejar de contestar al cuetionario *[It is quite probable that people could stop answering the questionnaire]* |
| --- | --- | --- | --- |

- Pensando en toda la población general del Ecuador: ¿piensas que algunas personas podrían no contestar este ítem de manera totalmente honesta? *[Thinking of the entire general population of Ecuador: ¿* *do you think some people might not answer this item honestly? ]]*

| Para nada / poco probable  *[Not at all / unlikely]* |  |  | Es bastante probable que las personas no contesten de manera totalemente honesta *[It is quite probable that people will not answer honestly]* |
| --- | --- | --- | --- |

**Ítem 4: Me he sentido triste** *[I have felt sad]*

- Pensando en toda la población general del Ecuador: ¿cuánto piensas que este ítem podría perturbar a algunas personas? *[Thinking of the entire general population of Ecuador: ¿ how much you think this item might upset some people? ]*

| Para nada / poco probable  *[Not at all / unlikely]* |  |  | Podría seriamente perturbar a algunas personas *[It could seriously disturb some people]* |
| --- | --- | --- | --- |

- Pensando en toda la población general del Ecuador: ¿piensas que este ítem haría que algunas personas dejen de contestar el cuestionario si lo encontrarán ahí? *[Thinking of the entire general population of Ecuador: ¿* *do you think this item might put someone off answering a questionnaire if they found it in there? ]*

| Para nada / poco probable  *[Not at all / unlikely]* |  |  | Es bastante probable que las personas pueda dejar de contestar al cuetionario *[It is quite probable that people could stop answering the questionnaire]* |
| --- | --- | --- | --- |

- Pensando en toda la población general del Ecuador: ¿piensas que algunas personas podrían no contestar este ítem de manera totalmente honesta? *[Thinking of the entire general population of Ecuador: ¿* *do you think some people might not answer this item honestly? ]*

| Para nada / poco probable  *[Not at all / unlikely]* |  |  | Es bastante probable que las personas no contesten de manera totalemente honesta *[It is quite probable that people will not answer honestly]* |
| --- | --- | --- | --- |

**Ítem 5: Me he sentido desesperanzado o sin esperanza** *[I have felt despairing or hopeless]*

- Pensando en toda la población general del Ecuador: ¿cuánto piensas que este ítem podría perturbar a algunas personas? *[Thinking of the entire general population of Ecuador: ¿ how much you think this item might upset some people? ]*

| Para nada / poco probable  *[Not at all / unlikely]* |  |  | Podría seriamente perturbar a algunas personas *[It could seriously disturb some people]* |
| --- | --- | --- | --- |

- Pensando en toda la población general del Ecuador: ¿piensas que este ítem haría que algunas personas dejen de contestar el cuestionario si lo encontrarán ahí? *[Thinking of the entire general population of Ecuador: ¿* *do you think this item might put someone off answering a questionnaire if they found it in there? ]*

| Para nada / poco probable  *[Not at all / unlikely]* |  |  | Es bastante probable que las personas pueda dejar de contestar al cuetionario *[It is quite probable that people could stop answering the questionnaire]* |
| --- | --- | --- | --- |

- Pensando en toda la población general del Ecuador: ¿piensas que algunas personas podrían no contestar este ítem de manera totalmente honesta? *[Thinking of the entire general population of Ecuador: ¿* *do you think some people might not answer this item honestly? ]*

| Para nada / poco probable  *[Not at all / unlikely]* |  |  | Es bastante probable que las personas no contesten de manera totalemente honesta *[It is quite probable that people will not answer honestly]* |
| --- | --- | --- | --- |

1. Elige, poniendo un visto, cuáles de las palabras/ emociones/experiencias que se encuentran en los cuadrados de la parte de abajo de cada ítem piensas que se acerca más a lo que pretende preguntar cada ítem. Elige una o varias según te parezca. Después señala con un círculo aquella que pienses que se acerca más al significado del ítem. *[Choose, by thinking in the box, which of the words/emotions/experiences found in the squares at the bottom of each item do you think is closer to what each item intends to ask. Choose one or several as you like. Then circle the one that you think is closest to the meaning of the item]*

**Ítem 1: Me he sentido irritado, molesto** *[I have felt irritated, annoyed]*

| Culpable *[guilty]* |  | deprimido*[depressed]* |  | desesperado*[desperate]* |  |
| --- | --- | --- | --- | --- | --- |
| asqueado*[nauseous]* |  | enfado*[angry]* |  | miserable*[miserable]* |  |
| avergonzado*[ashamed]* |  | irritable*[irritable]* |  | desdichado*[ufortunate]* |  |

**Ítem 2: Me he sentido infeliz** *[I have felt unhappy]*

| culpable*[guilty]* |  | deprimido*[depressed]* |  | desesperado*[desperate]* |  |
| --- | --- | --- | --- | --- | --- |
| asqueado*[nauseous]* |  | enfado *[angry]* |  | miserable*[miserable]* |  |
| avergonzado*[ashamed]* |  | irritable*[irritable]* |  | desdichado*[unfortunate]* |  |

**Ítem 3: Me he sentido tenso, ansioso o nervioso** *[I have felt tens anxious or nervous]*

| culpable*[guilty]* |  | deprimido*[depressed]* |  | desesperado*[desperate]* |  |
| --- | --- | --- | --- | --- | --- |
| asqueado*[nauseous]* |  | enfado *[angry]* |  | miserable*[miserable]* |  |
| avergonzado*[ashamed]* |  | irritable*[irritable]* |  | desdichado*[unfortunate]* |  |

**Ítem 4: Me he sentido triste** *[I have felt sad]*

| culpable*[guilty]* |  | deprimido*[depressed]* |  | desesperado*[desperate]* |  |
| --- | --- | --- | --- | --- | --- |
| asqueado*[nauseous]* |  | enfado *[angry]* |  | miserable*[miserable]* |  |
| avergonzado*[ashamed]* |  | irritable*[irritable]* |  | desdichado*[unfortunate]* |  |

**Ítem 5: Me he sentido desesperanzado o sin esperanza** *[I have felt despairing or hopeless]*

| culpable*[guilty]* |  | deprimido*[depressed]* |  | desesperado*[desperate]* |  |
| --- | --- | --- | --- | --- | --- |
| asqueado*[nauseous]* |  | enfado *[angry]* |  | miserable*[miserable]* |  |
| avergonzado*[ashamed]* |  | irritable*[irritable]* |  | desdichado*[unfortunate]* |  |
